# A study on the distribution of BK and JC polyomavirus in discarded donor kidneys

**DOI:** 10.3389/frtra.2025.1727407

**Published:** 2025-12-11

**Authors:** Wouter T. Moest, Aiko P. J. de Vries, Reshma A. Lalai, Hans J. Baelde, Jesper Kers, Els Wessels, Jason B. Doppenberg, Marten A. Engelse, Mariet C. W. Feltkamp, Joris I. Rotmans

**Affiliations:** 1 Division or Nephrology, Department of Internal Medicine, Leiden University Medical Center (LUMC), Leiden, Netherlands; 2 Leiden Transplant Center, Leiden University Medical Center (LUMC), Leiden, Netherlands; 3 Department of Pathology, Leiden University Medical Center (LUMC), Leiden, Netherlands; 4 Department of Pathology, Amsterdam UMC, University of Amsterdam, Amsterdam, Netherlands; 5 Department of Medical Microbiology & Infection Prevention, Leiden University Medical Center (LUMC), Leiden, Netherlands

**Keywords:** JC virus, BK virus, kidney transplantation, donor, renal tissue

## Abstract

**Introduction:**

BK polyomavirus (BKPyV) and JC polyomavirus (JCPyV) are thought to establish persistent, low-grade infections in the kidney. However, their specific intrarenal reservoirs remain unclear. To explore their localization and potential presence prior to transplantation, we analyzed different kidney regions from deceased donors.

**Method:**

Donor kidneys discarded for donation and subsequently designated for research purposes between November 2023 and October 2024 were included. For each kidney, cortex, medulla, pelvis, and ureter were sampled. These samples were analyzed using qPCR for the presence of JCPyV and BKPyV.

**Results:**

In total, 10 kidneys were analyzed with a total 72 samples taken from the cortex: *n* = 22, medulla: *n* = 22, renal pelvis: *n* = 14, and ureter: *n* = 14. All samples tested negative for BKPyV. JCPyV DNA was detected in 4 out of 10 kidneys. When analyzed by tissue type, positive samples were found in 6/22(27.3%) cortex, 6/22(27.3%) medulla, 4/14(28.6%) renal pelvis, and 4/14(28.6%) ureter samples. The cycle threshold (Ct) values did not show significant differences among the various regions within the kidney. Notably, JCPyV distribution within individual kidneys was markedly heterogeneous, with substantial variation in Ct-values within the same kidney.

**Conclusion:**

JCPyV DNA was detected in 40% of kidneys from deceased donors, with comparable detection rates across cortex, medulla, pelvis, and ureter, suggesting no clear tissue preference. However, within individual kidneys, the distribution and Ct-values varied considerably. BKPyV DNA was not detected in any sample. These findings support the hypothesis that JCPyV may be present prior to transplantation and potentially donor-derived. The potential role of JCPyV in kidney transplant recipients and its relationship with BKPyV warrant further investigation.

## Introduction

The BK Polyomavirus (BKPyV) and JC Polyomavirus (JCPyV) are human polyomaviruses belonging to the Polyomaviridae family. Both viruses are small (with an approximate diameter of 45 nm), non-enveloped with a circular double-stranded DNA. Their genomes are organized into three main regions: an early region encoding regulatory proteins, including the Large Tumor Antigen (LTag), a late region encoding structural proteins, and a non-coding control region (NCCR) that regulates replication and transcription. Primary infection with both JCPyV and BKPyV typically occurs without symptoms, likely via oral or respiratory transmission. The seroprevalence of these viruses is high in the general population, with BKPyV showing seroprevalance rates between 81% and 100% peaking at a younger age (20–29 years),whereas JCPyV seroprevalence ranges between 35 and 63.2% and tend to increase with age (1–4). In contrast, JCPyV shedding in the urine is more frequent and continuous in the general population (19%–41.2%), whereas BKPyV sheds less frequently (7–15.9%) and more occasionally (4, 5). After primary infection, it is thought that both viruses establish a persistent, low-grade infection in the kidney. However, their reservoir within the kidney has been scarcely studied, particularly regarding which specific kidney tissue serves as the primary reservoir for BKPyV and JCPyV (1).

In immunocompromised individuals BKPyV is primarily associated with kidney transplant patients, where it can cause BK Polyomavirus-associated nephropathy (BKPyVAN), often accompanied by high-level viremia. BKPyVAN is characterized by tubulointerstitial nephritis (TIN) and SV-40 antigen-positive staining in a kidney biopsy, where the staining identifies the LTag, but cannot discriminate between BKPyV and JCPyV (nor SV40 that belongs to the same genus *betapolyomavirus*). BKPyVAN can lead to graft damage and potentially graft loss (6). JCPyV, on the other hand, is primarily associated with progressive multifocal leukoencephalopathy (PML), a severe neurological disorder characterized by white matter lesions, permanent neurological damage and a high mortality rate of 20%–80% (7). However, rare cases have been reported in which JCPyV, like BKPyV, can cause nephropathy (8).

Notably, there is increasing evidence that BKPyV-related infections in kidney transplant patient may be donor-derived. For example, Wunderink et al. demonstrated a strong association between high BKPyV serotiters in the donor and an increased risk of BKPyV DNAemia in the kidney allograft recipient (9). Furthermore, Schmitt et al. reported that VP1 gene sequences detected in the urine of 20 donor-recipient pairs were completely identical between each donor and their corresponding recipient, further supporting the hypothesis of donor-derived transmission (10).

To gain insight into the distribution of these polyomaviruses within the kidney and to assess whether they are detectable prior to transplantation—potentially facilitating transmission to the recipient—we analyzed the localization of BKPyV and JCPyV-derived DNA in kidney tissues from discarded deceased donors by quantitative PCR (qPCR).

## Method

### Study population and design

All available post-mortal donor kidneys designated for research purposes were included in the study between November 2023 and October 2024. Kidneys were deemed unsuitable for transplantation based on macroscopic or histopathological findings assessed by the retrieval surgeon or pathologist (e.g., severe vascular atherosclerosis, parenchymal abnormalities, or procurement-related injury). The specific reason for discard was not systematically recorded or shared with the research team. None of the kidneys were rejected on virological or infectious grounds. Tissue samples were meticulously collected from 4 distinct, macroscopic intact, anatomic areas, including the cortex, medulla, pelvis, and proximal ureter. At the time of sampling, the largest possible tissue fragment per region was collected to ensure sufficient material for future analysis. Initially, 2 samples per region were collected, with one sample fixed in formaldehyde and the other snap-frozen using liquid nitrogen and stored at −80 °C. However, preliminary analysis of the first three kidneys revealed that viral DNA could not be detected in the majority of the samples prompting adjustment to the sampling protocol. To improve detection sensitivity, the number of cryopreserved samples per structure was increased. For the cortex and medulla, samples were taken from the upper pole, lower pole, and two from the mid-region. Additionally, two samples were collected from the pelvis and ureter. This adjustment was implemented for the last four kidneys.

Cryopreserved kidney tissue samples were sectioned into 10 µm slices and processed for DNA isolation and qPCR detection of JCPyV and BKPyV DNA. The BKPyV assay targeted a 90 bp fragment within the *VP1* gene using primers 440BKVs (5′-GAAAAGGAGAGTGTCCAGGG-3′) and 441BKVas (5′-GAACTTCTACTCCTCCTTTTATTAGT-3′) with probe 576BKV-TQ-FAM (5′-CCAAAAAGCCAAAGGAACCC-3′-BHQ1), as previously described (9, 11).

For JCPyV, primers 1339JCs (5′-GTCTCCCCATACCAACATTAGCTT-3′) and 1340JCas (5′-GGTTTAGGCCAGTTGCTGACTT-3′) with probe 1850JC-TQ-YAK (5′-TCTTTCCACTGCACAATCCTCTCATGAATG-3′) were used, amplifying a 130 bp fragment of the *large T antigen* gene (12). Assays were performed in multiplex with *phocid herpesvirus* (PhHV) as internal control to monitor extraction efficiency and PCR inhibition. Each qPCR run included a negative control and a BKPyV- or JCPyV-positive control sample to validate assay performance. Quantification of JCPyV was limited to semi-quantitative analysis, as a standardized quantitative assay for JCPyV is not routinely implemented in clinical diagnostics.

For histopathological evaluation, we selected tissue samples with the lowers JCPyV Ct-values, representing the highest viral load detected by qPCR. These samples were derived from formaldehyde-fixed, paraffin-embedded (FFPE) kidney tissue. Sections were prepared and stained for examination by light microscopy to assess histological features suggestive of active polyomavirus infection, including SV40 LTag staining.

### Statistical analysis

IBM SPSS statistics version 25 was used for statistical analysis. Donor characteristics (including age, sex, and cause of death) were summarized descriptively. Given the small cohort size (10 donors), no formal statistical significance testing was performed for these baseline characteristics. Age was reported as median and interquartile range (IQR). For comparisons of the proportion of JCPyV-positive samples across tissue types (cortex, medulla, renal pelvis, ureter), Fisher's exact test was applied. This test was chosen because several cell counts fell below 5, making the use of the chi-square test inappropriate. For the analysis of Ct-values, one-way ANOVA was used to evaluate whether viral loads differed significantly between tissue sites. Prior to ANOVA, the distribution of Ct-values was assessed for normality using the Shapiro–Wilk test, which confirmed that Ct-values were normally distributed (*p* > 0.05), supporting the use of parametric testing. All statistical tests were two-sided, and a *p*-value of <0.05 was considered statistically significant.

### Ethical considerations

Human deceased donor kidneys were procured through a multiorgan donor program. Organs were used only if the kidney could not be used for clinical kidney transplantation and if research consent was present, according to the local guidelines of the medical ethical committee of the Leiden University Medical Center and the Dutch Transplantation Foundation as the competent authority for organ donation in the Netherlands. Due to privacy regulations however, individual donor characteristics cannot be disclosed.

## Results

A total of 10 post-mortem donor kidneys from 10 different individuals were analyzed between November 2023 and October 2024. Across these 10 kidneys, a total of 72 tissue samples were collected, covering four distinct tissue types: cortex, medulla, pelvis, and ureter. The median age of the donors was 72.5 (IQR 4) years, and 3/10 (30%) donors were male. The primary cause of death was cardiovascular disease in 8/10 (80%) donors.

All 72 samples analyzed by qPCR were negative for BKPyV, while 20/72 (27.8%) samples, originating from 4/10 kidneys (40%), tested positive for JCPyV. From one kidney, 2/4 (50%) samples taken from the pelvis and ureter were positive. From another, 1/12 (8.3%) samples from the medulla tested positive. A third kidney had 5/12 (41.7%) positive samples, distributed across all four tissues. Finally, in one kidney, all 12 analyzed samples were positive for JCPyV.

The mean Ct-value for JCPyV-positive samples was 32.7 ± 4.3. In the kidney where all samples tested positive, Ct-values were slightly lower ranging from 24.02 to 35.29 (Table 1). There were no significant differences observed in the distribution of JCPyV-positive samples across the different tissue types, nor in the Ct-values between tissues (Table 2).

**Table 1:** Overview of qPCR positive samples for BKPyV and JCPyV across different kidney tissues.

Kidney ID	Tissue	Samples tested	BKPyV positive	JCPyV positive	JCPyV Ct values
2	Cortex	1	0/1	0/1	-
2	Medulla	1	0/1	0/1	-
2	Pyelum	1	0/1	1/1	32,36
2	Ureter	1	0/1	1/1	36,42
8	Cortex	4	0/4	4/4	31.55, 29.95, 29.76, 32.07
8	Medulla	4	0/4	4/4	35.29, 24.78, 24.02, 25.87
8	Pyelum	2	0/2	2/2	31.69, 32.77
8	Ureter	2	0/2	2/2	34.02, 32.05
9	Cortex	4	0/4	2/4	36.50, 34.07
9	Medulla	4	0/4	1/4	38.00
9	Pyelum	2	0/2	1/2	39.27
9	Ureter	2	0/2	1/2	37.04
10	Cortex	4	0/4	0/4	-
10	Medulla	4	0/4	1/4	36.88
10	Pyelum	2	0/2	0/2	-
10	Ureter	2	0/2	0/2	-

Table shows an overview of qPCR analysis for BKPyV and JCPyV across the different kidneys and tissues. N, number; Ct, cycle-threshold; JCPyV, JC polyomavirus; BKPyV, BK polyomavirus.

**Table 2:** Average presence of JCPyV and mean Ct values by tissue type.

Tissue	Cortex	Medulla	Pyelum	Ureter	Total	*p*-value
	(*N* = 22)	(*N* = 22)	(*N* = 14)	(*N* = 14)	(*N* = 72)	
qPCR detectable (*n*, %)	6 (27.3%)	6 (27.3%)	4 (28.6%)	4 (28.6%)	20 (27.%)	0.99*
Ct-value JCPyV (mean ± SD)	32.3 (2.6)	30.8 (6.6)	34.0 (3.5)	34.9 (2.3)	32.7 (1.0)	0.48**

N, number; qPCR, real-time polymerase chain reaction; Ct, cycle-threshold; SD, standard deviation; JCPyV, JC polyomavirus.

Table shows average presence of JCPYV and mean Ct values by tissue type. *P*-values were calculated using the

* Fisher's exact test or

** One-way ANOVA test. *P*-values < 0.05 were considered statistically significant.

With regard to the donors, there were no major differences in age, sex or cause of death between the JCPyV positive and JCPyV negative group. The median age in the JCPyV-negative group was 72.5 (IQR 8) years, compared to 69.0 (IQR 24) years in the JCPyV-positive group. The proportion of male donors was 1/4 (25%) in the JCPyV-positive group, compared to 2/6 (33.3%) in the JCPyV-negative group. The primary cause of death was cardiovascular disease in 5/6 (83.3%) JCPyV-negative donors and 3/4 (75%) JCPyV-positive donors. One JCPyV-negative donor (1/6,16.7%) died from pulmonary failure (COPD), while one JCPyV-positive donor (1/4,25%) died following trauma.

**Figure 1:**
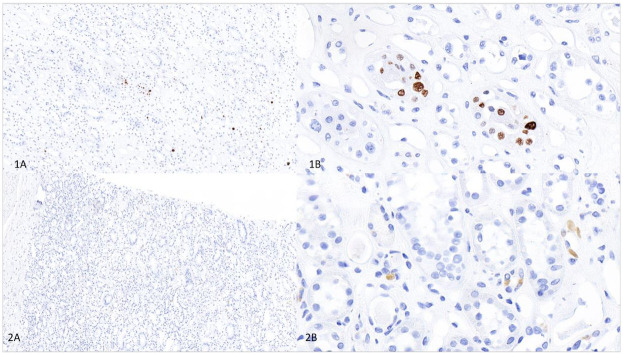
SV40 immunohistochemical staining of renal cortex and medulla from a JCPyV-positive donor kidney. Panel **(1A)** shows an overview of the medulla, and **(1B)** displays a higher magnification of the same area. Panel **(2A)** shows an overview of the cortex, and **(2B)** displays a higher magnification. SV40 immunohistochemistry revealed scattered nuclear positivity in a few tubular epithelial cells, primarily in the medulla.

Histopathological evaluation of the four different renal tissue types from the donor kidney with the highest JCPyV viral load (i.e., lowest Ct-value) showed no signs of inflammation. SV40 immunohistochemistry revealed scattered nuclear positivity in a few tubular epithelial cells, predominantly in the medulla, corresponding to the region with the lowest Ct-value. Although these nuclei appeared somewhat atypical, there were no accompanying features of tubulointerstitial nephritis (Figure 1).

## Discussion

In this study, the distribution of JCPyV and BKPyV in kidneys from discarded deceased donors was investigated. JCPyV was found in 40% of the kidneys, evenly distributed across various regions within the kidney, including the cortex, medulla, pelvis, as well as in the ureter. However, within individual kidneys, there was considerable variation in both the distribution and the range of viral load. Histologically, no signs of inflammation were observed in JCPyV-positive samples. In contrast, we could not detect BKPyV in any of the samples.

The renal reservoir of JCPyV in immunocompetent individuals has been investigated to a limited extend. Early studies by Chester et al.(1984) reported JCPyV in 3/31 kidneys, and 1/7 ureters from deceased individuals (13). Similarly, Boldorini et al. detected JCPyV in the bladder, renal pelvis, ureter, and kidney tissue in 53.5% of samples collected from 10 deceased individuals (14). These findings align with our results, and suggest that JCPyV does not exhibit a preference for a particular kidney tissue type.

Interestingly, unlike our findings, both Chester et al. and Boldorini et al. detected BKPyV in kidney tissue. Chester et al. found BKPyV in 10/31 kidneys, while Boldorini et al. detected BKPyV in 7/10 kidneys. The absence of BKPyV in our samples remains unexplained. Several possible explanations may account for this discrepancy. First, BKPyV may simply be less abundantly present in the kidney. This aligns with the observation that urinary shedding of BKPyV occurs less frequently than that of JCPyV. Second, methodological differences could play a role. Chester et al. used the Southern blot technique, this technique allows for analysis of substantially larger tissue volumes compared to our qPCR approach (usually 5–25x more DNA analysis), potentially increasing the chance of detection. In addition, it should be noted that the absence of BKPyV DNA in our samples may in part reflect sampling limitations, as BKPyV is known to exhibit a focal distribution within the kidney, leading to false-negative results (13). Importantly, the qPCR assay used in this study has recently been applied in an independent cohort of biopsy-proven BKPyVAN cases from our center, in which all diagnostic tissue samples tested clearly positive for BKPyV DNA. This confirms the analytical sensitivity of our assay and makes a technical failure unlikely. Third, it is possible that BKPyV is primarily present in the bladder, which was not sampled in this study. Supporting this, Boldorini et al. primarily detected BKPyV in the bladder (>60% of bladder samples tested positive), indicating that the bladder may serve as a preferred reservoir (13, 14). Lastly, epidemiological studies have shown that BKPyV subtype distribution, seroprevalence, and reactivation patterns vary substantially across demographic groups, which may further influence tissue detectability (15).

The detection of JCPyV in kidneys from deceased donors raises the question of whether transplant recipients could acquire JCPyV from the donor organ. Given that up to 50% of the general population is seronegative for JCPyV, a substantial proportion of kidney transplant recipients (KTRs) may still be susceptible to primary infection. Additionally, one could speculate that more virulent immune-escape variants of JCPyV exist, which could affect those previously exposed to the virus as well. This possibility is supported by several studies demonstrating post-transplant JCPyV seroconversion or a significant increase in JCPyV-specific IgG-titers in KTRs (16, 17). Moreover, metagenomic sequencing identified frequent transmission of JCPyV from kidney transplant donors to recipients, further reinforcing the hypothesis that JCPyV can be donor-derived (18). While there is evidence of donor derived transmission of BKPyV as well (9, 10), our inability to detect BKPyV does not support this in our study. The possibility of donor-derived JCPyV infection prompts consideration about its clinical relevance. While the prevalence of progressive multifocal leukoencephalopathy (PML) in KTRs is low, cases of JCPyV associated nephropathy (JCPyVAN) have been reported. Additionally, there is emerging evidence that JCPyV may play a role in BKPyVAN, potentially as a co-infection. Zhang et al. demonstrated that approximately 13% of KTRs with BKPyVAN also had JCPyV DNAemia, these patients experienced worse outcomes compared to those with BKPyVAN alone (19). This suggests that JCPyV and BKPyV co-infection could exacerbate the disease course in affected patients. Nemethova et al. demonstrated that polyomavirus large T-antigen transactivates E2F-regulated genes involved in S-phase induction, thereby pushing cells out of G1 and into S-phase—a state favorable for viral DNA replication. Based on this mechanism, one may hypothesize that strong JCPyV LTag expression could similarly render renal tubular cells more permissive to BKPyV replication in a co-infection scenario (20). In contrast Dolci et al, observed a potential protective role for JCPyV, they showed that kidney transplant recipients who received an organ from a donor with JCPyV DNAuria had a significantly lower risk of BKPyV DNAuria post transplantation (21). Moreover, additional studies found that the presence of JCPyV viruria in transplant recipients correlated with reduced BKPyV replication (22, 23). The potential for donor-derived JCPyV infection and its role in KTRs, particularly in relation to BKPyV, warrants further investigation (24, 25).

Lastly, in our dataset we observed substantial variation in Ct-values across different anatomical regions within the same kidney. One possible explanation for this heterogenous distribution, is that certain tissue areas contain only latent virus below the detection threshold, while other regions exhibit localized reactivation. In addition, local immune factors may contribute to this patchy pattern: regional differences in immune surveillance, tubular turnover, or inflammatory signaling may modulate the permissiveness of renal tissue for viral replication.

Such heterogeneity has important diagnostic implications. The focal distribution of JCPyV means that routine biopsy sampling may miss areas with active infection. Indeed, lessons from BKPyVAN—where including medullary sampling improves diagnostic yield—suggest that standard cortical biopsy strategies may underestimate JCPyV involvement in nephropathy (26).

This study has several limitations. First, we investigated JCPyV and BKPyV distribution in a small number of donor kidneys, limiting the generalizability of our findings. However, results of distribution within the kidney of JCPyV align with prior studies. Second, while we report Ct-values for JCPyV detection, it is important to recognize that this is a semi-quantitative measure of viral load, and caution should be exercised when interpreting these values. Third, we did not perform viral genome analysis, and therefore could not differentiate between archetype or rearranged NCCR variants. This limitation is relevant because NCCR rearrangements are known to enhance early gene promoter activity, leading to increased LTag expression and viral replication, which may in turn affect both the extent of intrarenal replication and the focal detectability in our samples. Finally, our sampling strategy involved analyzing only 10 µm sections per sample, representing only a small fraction of each kidney, which may have influenced the detection rates of both JCPyV and BKPyV.

In summary, we demonstrate that JCPyV is detectable in multiple renal structures (cortex, medulla, pelvis, and ureter) of deceased donor kidneys, whereas BKPyV was not detected. The role of JCPyV in kidney transplantation, including its potential impact on allograft function and its interaction with BKPyV, remains an area requiring further research.

## Data Availability

The raw data supporting the conclusions of this article will be made available by the authors, without undue reservation.
